# A rare cause of early repolarisation in an adolescent boy with chest pain: myocardial bridging

**DOI:** 10.5830/CVJA-2016-088

**Published:** 2017

**Authors:** Murat Deveci, Kadi Babaoğlu, Özlem Kayabey

**Affiliations:** Division of Paediatric Cardiology, Department of Paediatrics, Kocaeli University School of Medicine, Umuttepe-Kocaeli, Turkey; Division of Paediatric Cardiology, Department of Paediatrics, Kocaeli University School of Medicine, Umuttepe-Kocaeli, Turkey; Division of Paediatric Cardiology, Department of Paediatrics, Kocaeli University School of Medicine, Umuttepe-Kocaeli, Turkey

**Keywords:** myocardial bridging, early repolarisation, chest pain, adolescent

## Abstract

Early repolarisation is a common electrocardiographic (ECG) finding characterised by J-point and ST segment elevation ≥ 0.1 mV in two or more adjacent leads. The ECG pattern of early repolarisation is relatively common in asymptomatic subjects. Early repolarisation pattern may be seen in secondary conditions such as hypothermia, autonomic nervous system disturbances, cocaine abuse, hypercalcaemia and myocardial ischaemia. We present a case of an adolescent boy with chest pain and concurrent ST-segment elevation. Early repolarisation pattern was observed in the inferior leads of the ECG with increased troponin levels. He was shown to have myocardial bridging of the left anterior descending artery. The coronary anomaly was not associated with left ventricular hypertrophy. He was asymptomatic and the ECG changes normalised on the third day after admission. The patient was restricted from strenuous exertion and metoprolol was prescribed for prophylaxis.

## Introduction

Myocardial bridging is characterised by the systolic compression of a major coronary artery segment by the overlying myocardium. Although early reports considered it as a benign condition, it is currently known to be associated with myocardial ischaemia and infarction.^1^

Early repolarisation is defined as J-point and ST-segment elevation ≥ 0.1 mV in two or more contiguous leads. The ECG pattern of early repolarisation was initially described as a normal variant because of its occurrence in one to 13% of the general population. Athletes, particularly those participating in competitive sport, have a higher prevalence of early repolarisation. The judgment that early repolarisation was a benign finding devoid of clinical significance changed as studies determined an association between the presence of early repolarisation and an increased risk for arrhythmic death.^2^-^4^

We present a patient with chest pain who had ST-segment elevation (STE) and increased troponin levels and was found to have myocardial bridging. Early repolarisation pattern in the inferior leads was thought to result from ischaemia caused by the myocardial bridging, which adds uniqueness to the presentation.

## Case report

A 17-year-old boy was admitted to the emergency department with burning, exertional chest pain that persisted for two hours. His past medical history was unremarkable. He was a football player in the school team and trained regularly. The patient was not taking any medication and denied the use of illicit substances. No previous chest pain with or without exercise was described.

**Fig. 1.**
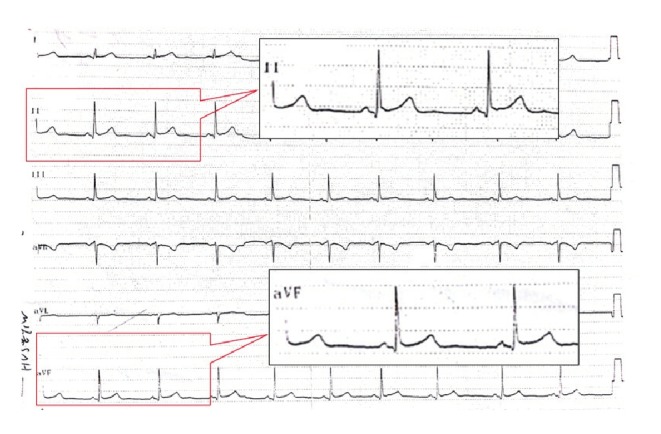
ECG on admission shows early repolarisation pattern in leads II, aVF.

His family history was unremarkable for hyperhomocysteinaemia, familial hyperlipidaemia and sudden death. Haemodynamic parameters and systemic examination on admission were negligible. The initial ECG showed normal sinus rhythm with early repolarisation pattern in the inferior leads (Fig. 1). Blood test values were all within the normal range, except troponin. On hospital admission, his troponin level was increased to 0.42 mg/ml and over two days at eight-hour intervals, his troponin level was undulant (0.42, < 0.01, 0.34, < 0.01, 0.13, < 0.01 ng/ml, respectively). Echocardiographic examination revealed no obvious heart disease, wall motion abnormalities or pericardial effusion.

**Fig. 2.**
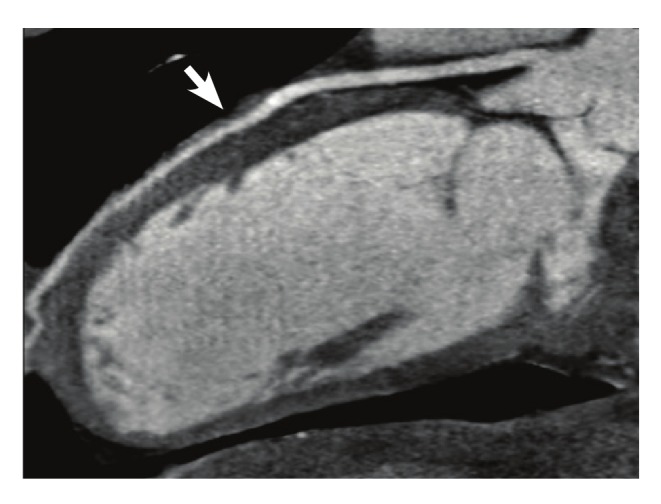
ECG-gated coronary CT angiography of the patient performed with 64-MDCT. A curved multi-planar reformat image shows a 12-mm bridging of the midsegment of the LAD. The arrow indicates the location of myocardial bridging. MDCT, multi-detector computed tomography; LAD, left anterior descending artery.

**Fig. 3.**
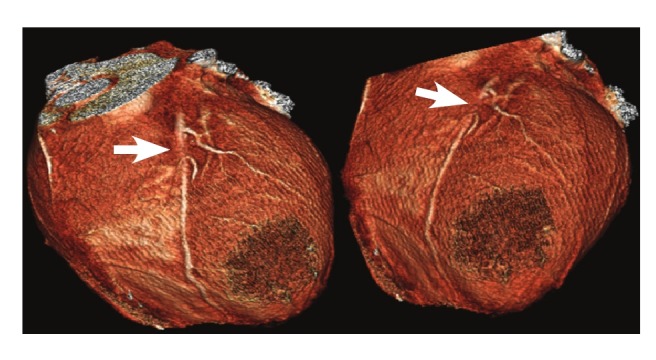
Three-dimensional volume-rendered image in which the myocardial bridging covers the upper middle segment of the LAD coronary artery. The arrow indicates the location of myocardial bridging.

As the patient’s chest pain was exertional, persistent and angina-like, in conjunction with the ECG findings and troponin levels, coronary artery imaging with multi-detector computed tomography (MDCT) was performed. Coronary myocardial bridging with a length of 12 mm was found in the middle tract of the left anterior descending artery (LAD) (Fig. 2, Fig. 3). For the assessment of myocardial perfusion, magnetic resonance imaging (MRI) was performed. Reduced perfusion of segments seven and 12, consonant with ischaemia, and weak contrast uptake, consonant with sub-endocardial infarct, were detected. These lesions matched the areas supplied by the bridged segment of the LAD that was defined by MDCT.

**Fig. 4.**
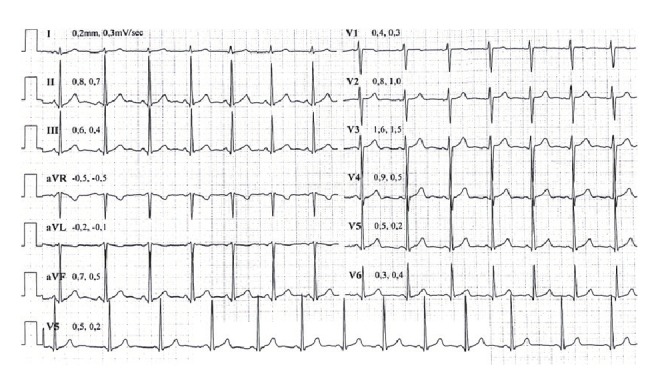
ECG at a baseline exercise-stress test on the seventh day of admission shows no abnormality.

The chest pain was relieved with aspirin and beta-blocker (metoprolol) therapy after the second day of admission. In the following days, the patient was asymptomatic, the cardiac enzymes were all within the normal range, and the initial ECG changes were attenuated (Fig. 4). A 24-hour Holter monitor demonstrated neither ectopic beats nor tachyarrhythmias. An exercise stress test, based on the Bruce protocol, revealed no ischaemic changes or arrhythmias.

The patient was restricted from strenuous exertion. Metoprolol was prescribed and the patient was discharged without any problems. We decided to remove the bridge surgically if he becomes symptomatic despite physical restriction and drug therapy. After the six-month follow up, the patient had no cardiac symptoms and his ECG remained normal.

## Discussion

Early repolarisation is defined as either a sharp, well-defined positive deflection or notch immediately following a positive QRS complex at the onset of the ST segment, or the presence of slurring at the terminal part of the QRS complex. Several population studies have estimated that the prevalence of early repolarisation ranges from five to 13% of persons.^5^,^6^

‘Early repolarisation pattern’ describes the patient with appropriate ECG findings in the absence of symptomatic arrhythmias. On the other hand, ‘early repolarisation syndrome’ applies to the patient with both appropriate ECG findings and symptomatic arrhythmias. Large population studies have shown that the presence of early repolarisation in the inferior leads on surface ECG is associated with an increased risk of death from cardiac causes as well as all-cause mortality.^2^,^3^ Our patient had early repolarisation pattern in the inferior leads with no documented arrhythmia.

Early repolarisation pattern may be seen in secondary conditions such as hypothermia, autonomic nervous system disturbances, cocaine abuse, antidepressant use, hypercalcaemia, neuropsychiatric disturbances, subarachnoid haemorrhage, metabolic diseases and cardiac diseases (acute coronary syndrome, myocardial ischaemia, hyper-vagotony, hypertrophic cardiomyopathy and pericarditis–myocarditis). Numerous benign and less life-threatening diseases such as early repolarisation, acute pericarditis and vasospastic angina can present with chest pain. ST-segment elevation on an electrocardiogram may occurin all these situations and many others, creating a diagnostic dilemma.

Originally considered to be a benign entity, recent reports suggest that bridging of the major coronary arteries may produce myocardial ischaemia, coronary thrombosis and myocardial infarction, as well as predispose the patient to atherosclerosis or sudden death.^7^,^8^ When symptoms occur in the presence of myocardial bridges, they are ischaemic in nature. The diagnosis of myocardial bridging of the LAD should be realised in patients who have exertional angina and myocardial perfusion defect but no coronary risk factors, especially those who are young, as in the presentation of our patient.

The prevalence of bridging has been reported to be around 25% in necropsy studies and around 2% in angiographic studies. Variation at autopsy may in part be attributable to the care taken in preparation and the selection of hearts. A higher prevalence has been observed in patients with hypertrophic cardiomyopathy and recipients of cardiac transplants.^1^,^9^,^10^ Our patient’s echocardiographic examination revealed no hypertrophy.

The site, length and severity of bridging and resultant coronary stenosis vary from patient to patient. Myocardial bridges are located at a depth of 1–10 mm with a typical length of 10–30 mm. Our patient had a 12-mm bridging segment of the LAD.

For the treatment of angina caused by myocardial bridging, beta-blockers and calcium channel blockers are preferred for negative chronotropic and inotropic effects. We administered metoprolol as a beta-blocker to our patient and he is presently asymptomatic. Surgical therapy is advised for patients with persistent symptoms and proven ischaemic changes, and forthose with high risk, such as ventricular arrhythmias, aborted sudden death, or non-fatal myocardial infarction. There are few reports of survival rates but, when studied, five-year survival ranges between 85 and 98%. Our patient was followed up with medical therapy.

The early repolarisation pattern is not always identified on routine ECG due to the intermittent nature of early repolarisation. For example, among 542 persons with baseline early repolarisation who underwent repeat ECG examination five years later, early repolarisation (≥ 0.1 mV) was not observed in approximately 20%.^2^,^5^ No systematic evaluation has been undertaken reporting the prevalence of concealed early repolarisation in the general population, and the clinical importance, if any, of concealed early repolarisation remains unclear. We believe that early repolarisation pattern in our patient was due to ischaemia caused by myocardial bridging and was not concealed.

## Conclusion

Differentiating ST-segment elevation caused by acute myocardial infarction from all other aetiologies, especially acute pericarditis– myocarditis, and early repolarisation, can be challenging. In ourpatient, anginal chest pain was thought to be due to myocardial bridging of the LAD artery, considering the possibility of a systolic narrowing of the coronary artery with subsequent ischaemia. Early repolarisation pattern in the inferior leads was deemed to result from ischaemia caused by myocardial bridging, which is the main point of this case. Ischaemia caused by myocardial bridging should also be considered in the differential diagnosis of early repolarisation in young patients.
